# Discussion on Syphilis

**Published:** 1906-05-05

**Authors:** 


### DISCUSSION ON SYPHILIS.

On Wednesday evening, April 11, the Hunterian Society devoted its deliberations to a "Discussion on Syphilis." The Society was particularly fortunate in having Mr. Jonathan Hutchinson to open the discussion, a fact sufficient in itself to ensure the success of the meeting. To every one present it was a very great pleasure to hear that great authority recount in his own lucid and vivid way some of his vast experience on the question of syphilis. Mr. Hutchinson pointed out that the death-rate from syphilitic affections was very much decreased of late years, and that the type of syphilis was now not nearly so virulent as it used to be. Improved methods of treatment were responsible for these facts, more especially the improvement in prolonging the period over which early treatment was carried out.

In the opinion of Mr. Hutchinson, of all the various methods of treatment none was so satisfactory to his mind as the administration, over a lengthened period, of the one-grain pill of hyd. c cret., three times a day, commencing as soon as the disease has been diagnosed.

Mr. Hutchinson briefly indicated the various conclusions that he had arrived at with regard to syphilis, and amongst others, that a man must not marry for two years after he has been infected, and then only if he has rigidly attended to treatment. That a woman must wait longer than a man before she marries. That treatment by mercury must be begun at once the disease is recognised. That it is possible to prevent and to anticipate secondary symptoms. That the treatment by intramuscular injections is very dangerous and should be reserved for patients under special conditions. That for all forms of gummatous growth in the tertiary stage iodide of potassium should be used internally and often gives permanent cure. That sometimes tubercular lesions become engrafted on syphilitic lesions.

Dr. Savage gave an account of syphilis and insanity, and showed in what ways syphilis might lead to the production of insanity. He described a syphilophobia which might be met with in the young man who, having exposed himself to the risk of infection, worked himself into the belief that he had actually got the disease. This might go to such an extreme that a suicidal tendency might develop. Again it might be met with in the older man who, having had an attack, imagined that he was looked on with horror and shunned for having been the victim of such a disease.

Dr. Savage gave some extremely interesting details with regard to the disease, "general paralysis of the insane." He showed how this disease almost invariably had syphilis as an ætiological factor in its causation, and to his mind it was necessary to add some other poison before the disease actually declared itself, *e.g.*, alcohol.

Dr. Purves Stewart followed with a most interesting paper on syphilitic disease of the nervous system. He pointed out that it was a curious thing that the nervous system was not attacked by the virus in the same way as other tissues were, but that it was really secondarily affected on account of its proximity to the lesion produced, *e.g.*, a gumma of the brain pressing on surrounding parts and thus producing symptoms.

The chief interest in Dr. Stewart's paper was his point on the diagnostic value to be attached to an examination of the cerebro-spinal fluid in cases of lesions in the central nervous system. He pointed out that whereas the normal fluid, after centrifugalisation, is found to contain only perhaps one or two lymphocytes in the field of the microscope, that in the case of, *e.g.*, Tabes Dorsalis, even in early stages before ataxy or any prominent symptoms have presented themselves, the cerebro-spinal fluid may contain as many as 250 lymphocytes to the field. Increase in the number of lymphocytes, says Dr. Stewart, is an extremely important point in the diagnosis of diseases affecting the central nervous system. In all doubtful cases examination of the cerebro-spinal fluid will be found an immense help in arriving at a correct diagnosis and as forming an excellent point for a differential diagnosis.

Then Dr. Hale White showed a most beautiful series of specimens illustrating syphilis of the viscera. He pointed out the vast importance of the fact that in certain cases there is a syphilitic constriction of one or other bronchus, producing diminished entry of air into corresponding lung with concomitant symptoms. Lung syphilis he showed to be very rare. Heart syphilis was a point on which Dr. White was extremely interesting, as he said that cases of sudden death were sometimes due to a syphilitic fibrosis of the inter-ventricular septum. This was not generally recognised and was often overlooked on account of the fact that its situation in the inter-ventricular septum rendered it liable to be overlooked. He had seen several cases of sudden death due to this condition.

Then Dr. White went on to liver affections and pointed out that gummatous affections of the liver were not nearly so common as they used to be, and he endorsed Mr. Hutchinson's opinion that the disease was certainly less virulent than in former days. Jaundice was seldom if ever produced by a syphilitic liver—a point in marked contrast to a malignant liver.

A short discussion followed, but as the hour was late it was resolved to postpone further discussion until after the meeting on April 25, when the subject will be again under discussion. The Society has every reason to congratulate itself on the success of the meeting, and those who were present have reason to thank the Society for affording them the opportunity to listen to such interesting accounts of this dire disease.

